# PROPDESC-Score-Validierung (PROPDESC-Val)

**DOI:** 10.1007/s00101-023-01371-4

**Published:** 2024-01-03

**Authors:** V. Guttenthaler, A. Kunsorg, A. Mayr, T. Hering, J. Menzenbach, M. Wittmann

**Affiliations:** 1 Klinik für Anästhesiologie und Operative Intensivmedizin, Universitätsklinikum Bonn, Venusberg-Campus 1, 53127 Bonn, Deutschland; 2 Institut für Medizinische Biometrie, Informatik und Epidemiologie, Universitätsklinikum Bonn, Bonn, Deutschland; 3 Klinik für Anästhesiologie, Intensivmedizin, Notfallmedizin und Schmerztherapie, Kreiskrankenhaus Mechernich GmbH, Mechernich, Deutschland

## Hintergrund und Hypothesen

Das postoperative Delir (POD) ist eine der häufigsten Komplikationen nach chirurgischen Eingriffen bei älteren Patienten mit einer Inzidenz von 11–51 % [1, 2]. Obwohl das POD als frühe, vorübergehende, postoperative Komplikation auftritt, kann es zu schwerwiegenden langfristigen Verschlechterungen führen, wie z. B. zu erhöhter Sterblichkeit, zu anhaltenden kognitiven Beeinträchtigungen sowie zur Einschränkung der Mobilität und der Selbstständigkeit [3]. Neben dem Alter und der Multimorbidität stellen sensorische, funktionelle und kognitive Beeinträchtigungen ein erhöhtes Risiko für POD dar [4]. Trotz der potenziell schwerwiegenden Schäden für die Patienten und der Belastung der Gesundheitsressourcen gibt es derzeit kein standardisiertes POD-Risiko-Screening in deutschen Krankenhäusern, obwohl auch die European Society of Anaesthesiology and Intensive Care (ESAIC) die Relevanz einer präoperativen Identifizierung von POD-Risikopatienten betont und sie zu einem Eckpunkt ihrer Leitlinie zum POD macht [3]. Die präoperative Vorhersage des POD-Risikos von Patienten bietet die Möglichkeit, Hochrisikopatienten rechtzeitig zu erkennen, um vorbeugende Maßnahmen einzuleiten. Chen et al. haben gezeigt, dass die im Modified Hospital Elder Life Program (mHELP) beschriebenen Maßnahmen das Auftreten des POD um 56 % reduzieren konnten [5].

Es gab einige Versuche, einen Risikoscore zur POD-Risiko-Detektion zu entwickeln, z. B. von Inouye et al. [6] und Kim et al. [7, 8], diese verwenden jedoch Parameter, die im Allgemeinen beim Prämedikationsbesuch nicht verfügbar sind. In den PROPDESC(Pre-Operative Prediction of Postoperative Delirium by Appropriate Screening)-Score [9] werden präoperativ verfügbare Ausgangsparameter einbezogen, ergänzt durch 2 sehr kurze kognitive Testfragen. Ziel von PROPDESC-Val (PROPDESC Score Validation) ist es, den intern am Universitätsklinikum Bonn entwickelten und validierten Score in unterschiedlichen Krankenhäusern Deutschlands extern als präoperatives POD-Risiko-Screening zu validieren.

## Details der Studie

PROPDESC-Val ist eine multizentrische, observatorische, investigatorinitiierte, prospektive Validierungsstudie, die in Krankenhäusern aller Versorgungsstufen in Deutschland durchgeführt wird (Einschlusskriterien: Alter ≥ 60 Jahre; mind. 1‑stündige elektive Operation, geplanter stationärer Aufenthalt in der Nacht nach der Operation, unterschriebene Einwilligungserklärung; Ausschlusskriterien: Patienten mit Sprachbarrieren, neurochirurgische Eingriffe, Betreuung, Demenz, Teilnahme an interventioneller POD-Studie; primärer Endpunkt: Validierung des PROPDESC-Scores in deutschen Krankenhäusern; sekundäre Endpunkte: Auswirkungen des POD auf die Dauer des Krankenhausaufenthalts und die Sterblichkeit im Krankenhaus). Zudem wird der Einfluss der Selbstversorgung, des präoperativen Alkoholkonsums und der Lagerung während der Operation auf das Auftreten eines POD untersucht.

Die Patienten werden an den ersten 5 Tagen nach der Operation vormittags mit CAM-ICU (Confusion Assessment Method for the Intensive Care Unit) auf der Intensivstation und mit 3D-CAM (3-Minute Diagnostic Interview for CAM-defined Delirium) auf Normalstation auf POD getestet (Tab. 1).

**Table 1:** 

	Visite 0	Visite 1	Visite 2	Visite 3	Visite 4	Visite 5	Entlasstag
	Screening	1. postop. Tag	2. postop. Tag	3. postop. Tag	4. postop. Tag	5. postop. Tag	
Ein‑/Ausschlusskriterien	x	–	–	–	–	–	–
Demographische Daten	x	–	–	–	–	–	–
Einwilligung	x	–	–	–	–	–	–
PROPDESC-Score	x	–	–	–	–	–	–
Delirtest	–	x	x	x	x	x	–
Operationsdaten	–	x	–	–	–	–	–
Dauer des Krankenhausaufenthalts	–	–	–	–	–	–	x

*postop.* postoperativ, *PROPDESC* Pre-Operative Prediction of Postoperative Delirium by Appropriate Screening

## Mitwirkung

Es werden weiterhin Krankenhäuser aller Versorgungsstufen in Deutschland gesucht. Falls Sie Interesse an der Mitarbeit bei PROPDESC-Val oder Fragen zur Studie haben, melden Sie sich gerne bei Prof. Dr. Maria Wittmann (Maria.Wittmann@ukbonn.de) oder Vera Guttenthaler (Vera.Guttenthaler@ukbonn.de).

## Statistik

Die Fallzahlberechnung basiert auf den Ergebnissen der PROPDESC-Studie. Die POD-Inzidenz lag hier bei gleichen Ein- und Ausschlusskriterien insgesamt bei 23,5 % bzw. bei 13 % in dem ausschließlich nichtkardiochirurgischen Kollektiv. Ergebnisse aus der Literatur legen nahe, dass für eine angemessene externe Validierung eines multivariablen prognostischen Scores etwa 200 „interessierende Ereignisse“ beobachtet werden sollten [10]. Unter der Annahme, dass in einigen der beteiligten Zentren keine Kardiochirurgie vertreten ist, rechnen wir konservativ mit einer Inzidenz von 13 %, was zu einer notwendigen Patientenzahl von mindestens *n* = 1550 Patienten führt. Mit dieser Stichprobengröße könnte man die AUC („area under the curve“) des PROPDESC-Scores mit einer Genauigkeit von ± 0,025 auf der Grundlage eines 95 %-Konfidenzintervalls schätzen. Um Ausfälle aufgrund fehlender POD-Tests zu kompensieren, die bis zu 35 % der Studienstichprobe ausmachen können, planen wir, *n* = 2400 Patienten einzubeziehen.

## Ethik

Die Studie wurde von der Ethikkommission der Medizinischen Fakultät des Universitätsklinikums Bonn am 04.05.2022 positiv bewertet (lfd. Nr.: 136/22). Das Universitätsklinikum Bonn übernimmt für jedes teilnehmende Zentrum das Einholen des lokalen Ethikvotums. Die Studie ist im Deutschen Register Klinische Studien unter der Nummer DRKS00028712 registriert.

Aus Qualitätsgründen werden in regelmäßigen Abständen Plausibilitätskontrollen der eingegebenen Daten (Database-Monitoring) durchgeführt. Dieser strukturierte Prozess ist bis zum Ende des Projekts geplant. Bei häufigen Unstimmigkeiten wird eine Vor-Ort-Kontrolle erwogen, um korrekte und valide Daten zu gewährleisten.

## Meilensteine

Seit Studienstart im November 2022 konnten 13 Kliniken in ganz Deutschland bis November 2023 bereits 1396 Patienten einschließen. Ende März 2025 soll die geplante Zahl von 2400 Patienten erreicht und die Studie beendet werden. Direkt im Anschluss werden die Daten ausgewertet und veröffentlicht.

## Studiengruppe/Expertise

- *Studienleiterin:* Prof. Dr. med. Maria Wittmann
- *stellvertretender Studienleiter:* Dr. med. Jan Menzenbach
- *Studienkoordination:* Vera Guttenthaler und Andrea Kunsorg, Klinik für Anästhesiologie und Operative Intensivmedizin, Universitätsklinikum Bonn
- *Statistiker:* Prof. Dr. Andreas Mayr, Institut für medizinische Biometrie, Informatik und Epidemiologie, Universitätsklinikum Bonn
- *Teilnehmende Zentren:* Uniklinik RWTH Aachen, Cura Krankenhaus Bad Honnef, Charité Berlin, Johanniter Krankenhaus Bonn, Universitätsklinikum Bonn, Klinikum Dortmund, Cellitinnen Krankenhaus Köln, RKH Orthopädische Kliniken Markgröningen, Kreiskrankenhaus Mechernich, LMU München, TU München, Klinikum Barmherzige Brüder Straubing, Universitätsklinikum Würzburg (vollständige Adressen siehe Zusatzmaterial in der Online-Version des Artikels)

Das Studienteam der Klinik für Anästhesiologie am Universitätsklinikum Bonn ist neben der Durchführung von eigenen Studien wie der Vorgängerstudie PROPDESC auch seit vielen Jahren als renommiertes Studienzentrum im nationalen und internationalen Bereich tätig. Ab Januar 2024 wird es die Funktion des Partnerinstituts des DGAI(Deutsche Gesellschaft für Anästhesiologie und Intensivmedizin)-Studienzentrums übernehmen.

### Infobox Link zur Studienbeschreibung der DGAI

https://www.dgai.de/forschung-preise/dgai-studienzentrum/dgai-gefoerderte-multizenterstudien.html 
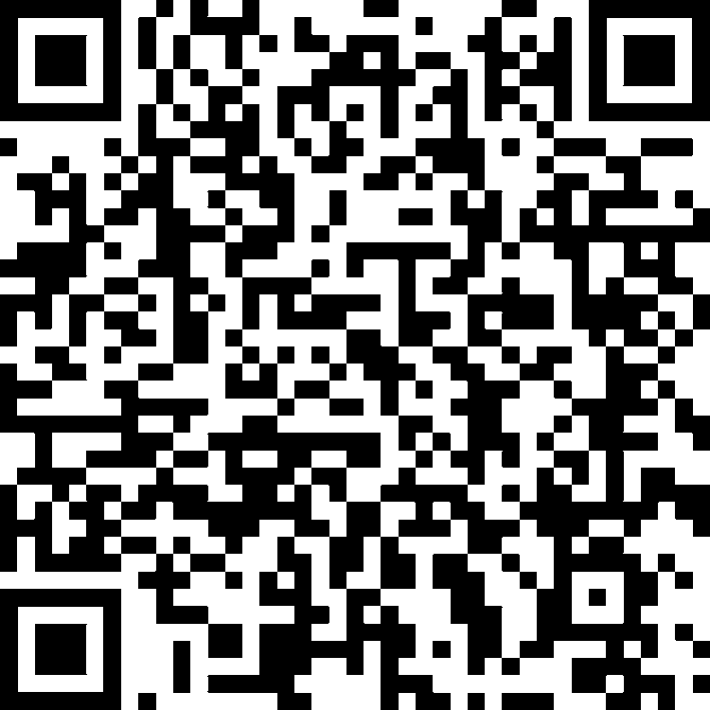


### Supplementary Information




