# Supplementary material for: PROPDESC Score Validation (PROPDESC-Val)
Source: Anaesthesiologie. 2024 Jan 3;73(1):56–9. [Article in German] doi: 10.1007/s00101-023-01371-4 (PMC10791728; doi:10.1007/s00101-023-01371-4)
Supplement: Supplementary file 1 [file 101_2023_1371_MOESM1_ESM.pdf]

Ergänzung zum Artikel **„PRä-Operative Vorhersage des postoperativen DEliriums durch geeignetes SCreening" (PROPDESC) Score-VALidierung (PROPDESC-Val)** von Guttenthaler V, Kunsorg A, Mayr A et al. (2023) in *Die Anaesthesiologie*.
Beitrag und Zusatzmaterial stehen Ihnen auf www.springermedizin.de zur Verfügung. Bitte geben Sie dort den Beitragstitel in die Suche ein.

Vollständige Liste aller an PROPDESC-Val beteiligten Studienzentren mit Projektleiter in alphabetischer Reihenfolge:

1. Aachen

Dr. med. Julia Wallqvist
Dr. med. Linda Grüßer
Dr. med. Sebastian Ziemann
Klinik für Anästhesiologie
Universitätsklinikum Aachen
Pauwelsstraße 30
52074 Aachen

2. Bad Honnef

PD Dr. med. Stefan Wirz
Nadine Laabs
Chefarzt der Abteilung für Anästhesie, Interdisziplinäre Intensivmedizin, Schmerzmedizin/Palliativmedizin – Zentrum für Schmerzmedizin, Weaningzentrum
Cura Krankenhaus
Schülgenstr. 15
53604 Bad Honnef

3. Berlin

Univ.-Prof. Dr. med. Sascha Treskatsch
Dr. med. Lennart Junge
Phillipp Brandhorst
Nicole Vogel
Klinikdirektor
Charité - Universitätsmedizin Berlin
Klinik für Anästhesiologie m. S. operative Intensivmedizin
Charité Campus Benjamin Franklin
Hindenburgdamm 30
12203 Berlin

4. Bonn

Prof. Dr. med. Maria Wittmann

Dr. med. Gregor Massoth
Alma Puskarevic
Vera Guttenthaler
Alexa Zimmermann
Vesna Vuijic
Natalie Dahmen
Klinik für Anästhesiologie und Operative Intensivmedizin
Universitätsklinikum Bonn
Venusberg-Campus 1
53127 Bonn
Tel.: +49 (0)228 287 14134
Maria.Wittmann@ukbonn.de

Prof. Dr. med. Georg Baumgarten
Dr. med. Frank Hentschel
Klinik für Anästhesiologie, Operative Intensivmedizin und Schmerzmedizin
Johanniter Krankenhaus Bonn
Akademische Lehrkrankenhäuser der Rheinischen Friedrich-Wilhelms-Universität Bonn
Johanniterstraße 3-5
53113 Bonn

5. Dortmund

Prof. Dr. med. Richard Ellerkmann
Dr. Karin Wenning
Antje Schüpphaus
Andrea Grebeldinger
Klinikum Dortmund gGmbH
Beurhausstr. 40
44137 Dortmund
www.klinikumdo.de

6. Köln

Prof. Dr. med. Stefan Weber
Martin Rößler
Heilig Geist-Krankenhaus Köln
Graseggerstr. 105
50737 Köln

7. Markgröningen

PD Dr. med. Se-Chan Kim
Mauricio Franco
Zentrum für Anästhesiologie, Perioperative Medizin und Schmerztherapie
Orthopädische Klinik Markgröningen
Kurt-Lindemann-Weg 10
71706 Markgröningen

8. Mechernich

Prof. Dr. med. Rudolf Hering

Theresa Hering
Klinik für Anästhesiologie, Intensivmedizin, Notfallmedizin und Schmerztherapie
Kreiskrankenhaus Mechernich GmbH
Akademisches Lehrkrankenhaus der Universität Bonn
St.-Elisabeth-Str. 2-6
53894 Mechernich

9. LMU München

PD Dr. med. Thomas Saller
Dr. Anne-Maire Just
Dr. Margret Rudy
Mahmoud Almaghrabi
Daniel Rauschenbach
Klinikum der Universität München
Klinik für Anästhesiologie
Marchioninistr. 15
81377 München

10. TU München

PD Dr. med. Stefanie Pilge
Dr. med. Svenja Letz
Susanne Maluche
Linda Mitteregger
Wilfried Arnoldi
Pia Feddersen
Klinik für Anästhesiologie und Intensivmedizin
Klinikum rechts der Isar
der Technischen Universität München
Ismaninger Str. 22
81675 München

11. Klinikum Straubing

Prof. Dr. med. Matthias Jacob
Samuel Willkomm
Klinik für Anästhesiologie, Operative Intensivmedizin und Schmerzmedizin

BARMHERZIGE BRÜDER
Klinikum St. Elisabeth Straubing GmbH
St.-Elisabeth-Str. 23
94315 Straubing

12. Würzburg

Dr. med. Dr. rer. nat. Benedikt Schmid
Tri Dinh
Prof. Dr. med. Patrick Meybohm

Klinik und Poliklinik für Anästhesiologie, Intensivmedizin, Notfallmedizin und Schmerztherapie
Universitätsklinikum Würzburg
Oberdürrbacher Str. 6
97078 Würzburg
